# Functional Properties of Probiotics in Food Sources

**DOI:** 10.3390/foods13162548

**Published:** 2024-08-15

**Authors:** Jiajia Song

**Affiliations:** College of Food Science, Southwest University, Chongqing 400715, China; jiajias@swu.edu.cn

## 1. Introduction

Probiotics are live microorganisms that, when consumed in sufficient quantities, offer numerous health benefits to the host. Dairy products, fermented foods, fruits, and vegetables are prime sources of these beneficial microbes. Research has shown that probiotics from food sources exhibit various bioactivities, such as improving gut health, mitigating food protein allergies, and enhancing exercise performance [1,2,3]. They also play a role in preventing disorders beyond the intestines, including metabolic disorders [4], hyperuricemia [5], polycystic ovarian syndrome [6], and neurodegenerative diseases [7]. In addition, prebiotics are bioactive compounds that are either added to foods or naturally present in them, serving as substrates for specific gut microorganisms and providing health benefits [8]. Synbiotics, which combine probiotic bacteria with prebiotic ingredients, exhibit even stronger bioactivities, such as reducing lipid accumulation and alleviating autism spectrum disorder-like behaviors, compared to probiotics alone [9,10]. Postbiotics are defined as preparations of non-viable microorganisms and/or their cellular components that provide health benefits to the host, with obesity, immune system function, and microbiota being key targets [11,12]. Probiotics are also widely used in fermented foods. For instance, *Lactobacillus casei* Zhang has been found to enhance the properties of stirred yogurt by increasing gel strength and exopolysaccharide content and improving various technological aspects [13]. Similarly, *Lactobacillus paraplantarum* LS-5 has been reported to boost the antioxidant activity of fermented sauerkraut [14].

This Special Issue, titled “Functional Properties of Food Source Probiotics”, in *Foods* includes eleven articles in total: ten research articles and one review article. The content is primarily focused on the following three areas: the biological functions of probiotics, postbiotics, and prebiotics; the characteristics and flavors of fermented foods, such as fruit wines, fruit juices, and yogurts; and the safety evaluation of probiotic strains.

## 2. An Overview of Published Articles

Tan et al. isolated *Lactiplantibacillus plantarum* HFY11 (LP-HFY11) from naturally fermented yak yogurt and investigated its effects on oxazolone-induced colitis in mice [Contribution 1]. Their study shows that LP-HFY11 significantly alleviates colitis symptoms and modulates inflammation- and immunity-related factors in the colon and serum. Additionally, LP-HFY11 was found to influence gut microbiota composition. These findings suggest that LP-HFY11 has potential as a probiotic and may be effective in treating oxazolone-induced colitis. In a separate study, Lee et al. demonstrated for the first time that *Lactiplantibacillus brevis* GKEX, sourced from douchi, in combination with resistance exercise training, can enhance muscle mass, thickness, strength, and power, while reducing body fat percentage over a six-week period in a double-blind randomized trial [Contribution 2]. Niu et al. explored the effects of heat-killed *Bifidobacterium longum* BBMN68 in pasteurized yogurt on alleviating mugwort pollen-induced allergic inflammation. Their findings indicate that the pasteurized yogurt containing heat-killed BBMN68 reduces allergic airway inflammation by maintaining the Th1/Th2 immune balance and altering gut microbiota structure and function [Contribution 3]. Similarly, Shiu et al. examined the effects of various inactivated forms of *Lactobacillus gasseri* HM1, with or without recombinant lactoferrin (LF), on metabolic disorders in obese mice [Contribution 4]. Their results suggest that different deactivation methods influence the efficacy of probiotics in improving metabolic indices. Specifically, sonication-killed HM1 (SK-HM1) is most effective in reducing inflammation in perirenal and epididymal fat tissues, while SK-HM1/LF shows significant improvement in glucose tolerance, highlighting the potential benefits of recombinant LF for glucose metabolism. Additionally, the exopolysaccharide from *Lactiplantibacillus plantarum* HMX2, isolated from Chinese northeast sauerkraut, was supplemented in the diet of juvenile turbot. This addition enhances growth performance, boosts antioxidant, digestive, and immune-related enzyme activities, improves survival rates, and regulates the intestinal microbiota, indicating its positive impact on juvenile turbot cultivation [Contribution 5]. Deng et al. reviewed the mechanisms by which probiotics and their components and metabolites mitigate colon cancer, providing a comprehensive overview of their effects on the disease [Contribution 6]. Furthermore, water-insoluble polysaccharide from Poria cocos, a type of prebiotic, was found to restore intestinal barrier function, increase short-chain fatty acids, reduce inflammatory cytokines, and modify gut microbiota structure, effectively alleviating antibiotic-associated diarrhea in mice [Contribution 7].

In fruit wine production, *Saccharomyces cerevisiae* is commonly used, but the application of non-Saccharomyces strains can introduce new and desirable flavors. Compared to mulberry wine fermented solely with *S. cerevisiae*, co-fermentation with *P. kluyveri* significantly reduces the levels of 2-methylpropyl acetate, allyl isothiocyanate, ethyl crotonate, isobutyl propanoate, butyl 2-methylbutanoate, alcohols, aldehydes, ketones, and organic acids, suggesting that *P. kluyveri* can create a novel flavor profile for mulberry wine [Contribution 8]. Balmori et al. investigated five pomelo cultivars for probiotic beverage production using *Lacticaseibacillus paracasei* and identified Tubtim Siam as the most suitable cultivar due to its high *L. paracasei* survival, antioxidant properties, bile acid binding, lipase activity inhibition, and cholesterol micellization disruption [Contribution 9]. He et al. studied the effects of konjac glucomannan (KGM) on the growth of *Lactiplantibacillus plantarum* SHY130 and evaluated the synbiotic combination of *L. plantarum* SHY130 and KGM on the physicochemical, antioxidant, and sensory properties of stirred yogurt. Their research offers new insights into developing synbiotic yogurt products [Contribution 10].

To be considered a probiotic, a bacterial strain must have a well-defined safety profile. Ma et al. assessed the safety of *Akkermansia muciniphila* PROBIO, demonstrating that it lacks mutagenic or clastogenic properties, does not spread antibiotic resistance genes, shows no toxicological effects in acute and sub-chronic toxicity studies, lacks potential virulence factors or pathogenic properties, and its moxifloxacin resistance does not transfer. These findings confirm the safety of *A. muciniphila* PROBIO for food applications [Contribution 11].

## 3. Conclusions

The articles in this Special Issue reveal the effects and mechanisms of probiotics, postbiotics, and prebiotics in improving constipation, diarrhea, colon cancer, athletic performance, allergic reactions, metabolic syndrome, and animal growth, as well as their safety. Additionally, some strains were explored for their applications in enhancing the quality and flavor of fermented foods. The findings in this Special Issue emphasize the significance of probiotics in food sources and further encourage research development in this area. In the future, research on the action mechanisms of probiotics should focus on identifying key molecular targets and fundamental substances, while the evaluation of effects in human trials should also be considered.
